# Evaluation of circulating tumor DNA as a biomarker in pancreatic cancer with liver metastasis

**DOI:** 10.1371/journal.pone.0235623

**Published:** 2020-07-02

**Authors:** Yasunori Uesato, Naoki Sasahira, Masato Ozaka, Takashi Sasaki, Mitsuhisa Takatsuki, Hitoshi Zembutsu

**Affiliations:** 1 Project of Development of Liquid Biopsy Diagnosis, Cancer Precision Medicine Center, Japanese Foundation for Cancer Research, Tokyo, Japan; 2 Department of Digestive and General Surgery, Graduate School of Medicine, University of the Ryukyus, Okinawa, Japan; 3 Department of Gastroenterology, Cancer Institute Hospital, Japanese Foundation for Cancer Research, Tokyo, Japan; Seoul National University College of Pharmacy, REPUBLIC OF KOREA

## Abstract

Pancreatic cancer is an aggressive, solid tumor, with a grave prognosis. Despite surgical treatment in patients with pancreatic cancer, the rate of recurrence is high. In addition, although tumor biomarkers are frequently used to confirm advanced pancreatic cancer, this is not accurate and the biomarkers currently used cannot indicate prognosis. This study sought to evaluate circulating tumor DNA as a tumor biomarker to prognosticate pancreatic cancer. Patients with advanced pancreatic cancer and liver metastasis (N = 104) were included, and blood samples were collected from all patients. The mutant allele frequency was measured using amplicon-based deep sequencing on a cell-free DNA panel covering 14 genes with > 240 hot spots. In patients with advanced pancreatic cancer, 50% (N = 52) had detectable ctDNA levels, with *TP53* (45%, N = 47) and *KRAS* (42.3%, N = 44) mutations the most common. Patients with detectable circulating tumor DNA levels also had significantly worse overall survival and progression free survival than ctDNA negative patients (8.4 vs 16 months, P<0.0001 for overall survival; 3.2 vs 7.9 months, P<0.0001 for progression-free survival). In a multivariate analysis, ctDNA status was independently associated with overall survival and progression-free survival (HR = 3.1, 95%CI = 1.9–5.0, P<0.0001; HR 2.6, 95%CI = 1.7–4.0, P<0.0001, respectively). Moreover, circulating tumor DNA significantly correlated with a higher number of liver metastases, the presence of lung and/or peritoneal metastases, tumor burden, and higher carbohydrate antigen 19–9 levels. This study supports the use of circulating tumor DNA as an independent prognostic marker for advanced pancreatic cancer.

## Introduction

Pancreatic cancer is one of the deadliest diseases, with a very poor prognosis. Patients with metastatic pancreatic cancer have a 5-year overall survival (OS) rate of only 2% [1], and fewer than 5% of patients with advanced pancreatic cancer who receive chemotherapy are expected to survive 5 years [2–5]. The prognosis remains poor despite improvements in treatment, particularly the introduction of new chemotherapies and surgical techniques, especially as cancer is usually detected in advanced stages. Patients with advanced pancreatic cancer must be monitored often to evaluate tumor burden and response to treatment. Serum protein biomarkers, such as carcinoembryonic antigen (CEA) and carbohydrate antigen 19–9 (CA19-9), are frequently measured, but these biomarkers do not accurately predict prognosis [6]. As such, a stringent yet minimally invasive biomarker that measures patient status with high sensitivity and specificity is urgently needed.

Recently, circulating tumor DNA (ctDNA), which is cell-free DNA (cfDNA) derived from a malignant tumor, has been suggested as a biomarker in various cancers. cfDNA increases during various physiological changes caused by factors including inflammation, smoking, and infection, but also increases in cancer patients [7, 8]. cfDNA is mainly caused by apoptosis and necrosis, and apoptosis is greatly increased in tumor masses due to the overgrowth of cancer cells and rapid cell turnover. Thus, cell debris normally phagocytosed by macrophages cannot be completely removed, and instead accumulates and is released into the blood circulation [9–11]. ctDNA has been recently confirmed as an early marker for treatment response and disease progression in patients with metastatic colorectal cancer and breast cancer [12, 13]. In pancreatic cancer, ctDNA is considered to be a useful biomarker for the risk of postoperative recurrence and for evaluating patient responses to chemotherapy [14, 15]. ctDNA levels may also be clinically useful for the detection of pancreatic cancer recurrence during postoperative follow-up [16–18]. Although several studies have investigated the utility of ctDNA as a biomarker in patients with pancreatic cancer [16, 19], its ability to predict prognosis is still unclear. Here, we sought to examine the utility of ctDNA as a biomarker in patients with advanced pancreatic cancer and liver metastasis.

## Materials and methods

### Patients

Blood samples were collected from 106 patients with advanced pancreatic cancer and liver metastasis who were undergoing follow-up at the Cancer Institute Hospital, Japanese Foundation for Cancer Research from February 2017 to August 2018. Two patients were excluded because of the presence of cancer in other organs, leaving a final cohort of 104 patients with advanced pancreatic cancer and liver metastasis. A power calculation was not conducted in this study because the study was designed with a relatively large sample size in previous reports. A specific course of treatment was not a requirement for enrollment in the study (Table 1). Computed tomography (CT) imaging was performed for all included patients to examine the degrees of pancreatic cancer progression, the number of liver metastases, and other metastases. The study was approved by the Institutional Review Board of the Japanese Foundation for Cancer Research (Tokyo, Japan), and written informed consent was obtained from all patients.

**Table 1:** Patient demographics and clinical characteristics.

Characteristics	Total (N = 104) No. of patients (%)
Age at enrollment, y	
Median [range]	64 [34–90]
Gender	
Male	67 (64.4)
Female	37 (35.6)
Treatment line at sampling	
None	1 (0.96)
1^st^ line	38 (36.5)
2^nd^ line	37 (35.6)
3^rd^ line	22 (21.2)
4^th^ line	6 (5.8)
Chemotherapy regimen at sampling	
GnP	61 (58.7)
mFFX	34 (32.7)
S-1	3 (2.9)
GEM	3 (2.9)
GE	2 (1.9)
None	1 (0.96)
Pancreatic location of tumor	
Head	56 (53.8)
Body or tail	48 (46.2)
Surgery at time of blood sample	
Yes	23 (22.1)
No	81 (77.9)
Metastatic site	
Liver	104 (100)
Lymph node	75 (72.1)
Lung	19 (18.2)
Peritoneum	11 (10.6)
Others	7 (6.7)
Tumor marker	
CEA, ng/ml median [range]	6.55 [1–610.8]
CA19-9, U/mL median [range]	1280 [2–≥50000]

CEA, carcinoembryonic antigen; CA19-9, carbohydrate antigen 19–9; GnP, a combination of gemcitabine and nab-paclitaxel; mFFX, FOLFIRINOX modified regimen; FOLFIRINOX, a combination of folinic acid and fluorouracil and irinotecan with oxaliplatin; S-1, a combination of tegafur, gimeracil and oteracil potassium; GEM, gemcitabine; GE, a combination of gemcitabine and erlotinib.

### Blood sampling and ctDNA isolation

Blood was collected into EDTA tubes following the manufacturer’s instructions. To obtain plasma for analysis, blood samples were centrifuged for 10 min at 2,000×*g* at 4°C, then the supernatant was centrifuged a second time for 10 min at 16,000×*g* at 4°C to remove cellular debris. cfDNA was extracted from the plasma samples using the MagMax Cell-Free DNA Isolation Kit (Life Technologies; Thermo Fisher Scientific, Inc.) according to the manufacturer’s instructions. The cfDNA fraction was obtained from plasma samples using magnetic beads with three washing and rebinding cycles. cfDNA concentrations were determined using a Qubit dsDNA HS Assay kit (Life Technologies; Thermo Fisher Scientific, Inc.). cfDNA size distributions were analyzed using an Agilent 2100 Bioanalyzer (Agilent Technologies, Inc.).

### Sequencing

An Oncomine Colon cfDNA Assay (Thermo Fisher Scientific) was used to generate libraries from the cfDNA, following the manufacturer’s instructions. The purity and concentration of the library were assessed using the Qubit dsDNA HS Assay kit and the Agilent 2100 Bioanalyzer, respectively. Unique index tags were added to each DNA fragment during library preparation. The Ion Chef System and the Ion 530 Kit-Chef were used for template preparation, followed by sequencing on the Ion S5 system using Ion 530 chips. A six-plex library pool was applied to the Ion 530 chip. We used a cfDNA panel covering 14 genes and 48 amplicons, including 240 hot spots (SNVs and short indels). The genes included in the panel were *KRAS*, *TP53*, *APC*, *FBXW7*, *GNAS*, *MAP2K1*, *CTNNB1*, *ERBB2*, *PIK3CA*, *BRAF*, *EGFR*, *SMAD4*, *NRAS*, *and AKT1*. Clean reads were mapped to the human reference genome (hg19). A variant caller was used to filter and call mutations in targeted regions of each gene [20, 21]. The limit of detection for each variant (mutant allele frequency [MAF]) was 0.15%. The average coverage ranged from 20,000x to 50,000x.

### Statistical analysis

Chi-squared tests were used for comparisons of categorical variables (such as gender) and Mann–Whitney U tests were used for comparisons of continuous variables (such as age) between the ctDNA-positive and -negative groups. In addition, we analyzed the relationships between ctDNA and CT findings and relevant blood markers, including CA19-9 and CEA. The response rate was evaluated using the criteria set out in the Response Evaluation Criteria in Solid Tumors (RECIST) version 1.1. Survival was estimated using Kaplan–Meier curves [22, 23]. Correlations between clinical outcome and ctDNA markers were assessed using the log-rank test. Overall survival (OS) was calculated from the date of blood sampling to the date of death from any cause. Progression-free survival (PFS) was calculated from the date of blood sampling to the date of disease recurrence or progression, or death from any cause. The Cox hazard regression method was used to identify independent risk factors for OS and PFS. For all statistical analyses, two-tailed *P* values of 0.05 or less were considered statistically significant. All statistical analyses were performed using BellCurve for Excel version 3.10.

## Results

### Patient characteristics

Patient characteristics are presented in Table 1. The median age at the time of blood sampling was 64 years (range, 34–90 years), and 67 patients were male (64%). First, second, or third line chemotherapy was administered to 97 (93%) of the patients at the time of blood sampling. Only one patient did not receive chemotherapy either before or after blood sampling. Most (95/104; 91.4%) of the patients were treated with nab-paclitaxel added to gemcitabine (GnP) or a modified combination of three chemotherapeutic agents: fluorouracil, oxaliplatin, and irinotecan (mFFX). Surgery was performed before blood sampling for 21 patients and after blood sampling in two patients because of the disappearance of liver metastases following chemotherapy. Aside from the liver, the lymph node was the most frequent site of metastasis (72.1%), followed by the lung (18.2%) and peritoneum (10.6%). Median levels of CEA and CA19-9 at the time of sampling were 6.55 ng/ml and 1280 U/mL, respectively.

### Somatic mutations and frequency

**Fig 1:**
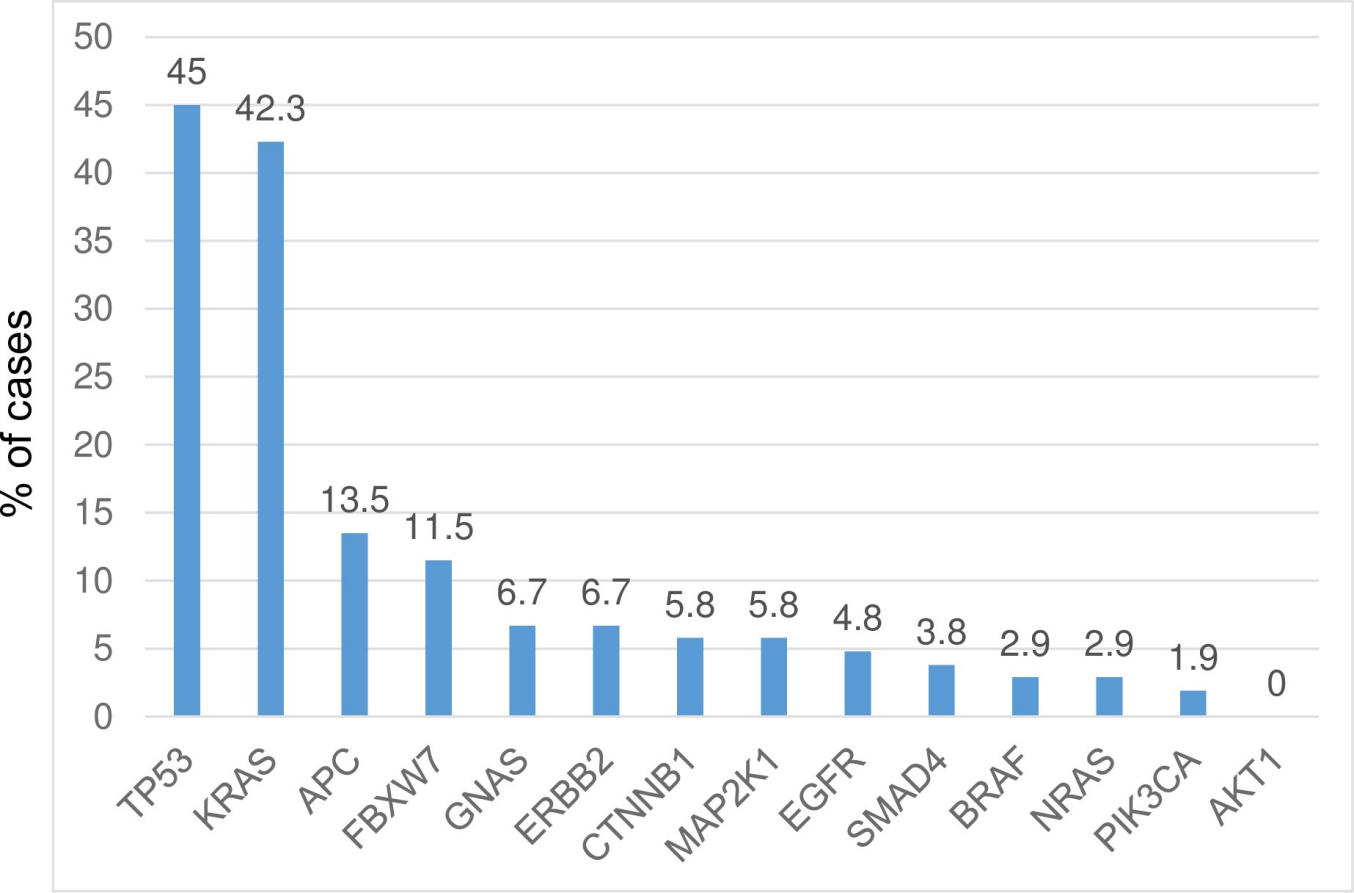
Frequencies of mutated genes in plasma. Distribution of somatic mutations in a panel of 14 genes in cfDNA from 104 pancreatic cancer patient plasma samples. One or more mutations were detected in all genes on the panel except *AKT1*.

Of the 104 patients enrolled in this study, half (52/104; 50%) had somatic mutations in one or more of 13 of the 14 genes in the panel (*TP53*, *KRAS*, *APC*, *FBXW7*, *GNAS*, *ERBB2*, *CTNNB1*, *MAP2K1*, *EGFR*, *SMAD4*, *PIK3CA*, *BRAF*, and *NRAS*); only one gene (*AKT1*) had no instances of mutation in this cohort (S1 Table). The frequency of mutations is shown in Fig 1. Mutations in *TP53* and *KRAS* were the most frequently detected (45% [47/104] and 42.3% [44/104] patients, respectively). *KRAS p*.*G12D*, *p*.*G12V*, *p*.*12R*, and other gene mutations were detected in 23, 15, 3, and 3 cases, respectively. *APC* and *FBXW7* were also frequently mutated (13.5% [14/104] and 11.5% [12/104] patients, respectively). Mutations in *GNAS* (6.7%), *ERBB2* (6.7%), *CTNNB1* (5.8%), *MAP2K1* (5.8%), *EGFR* (4.8%), *SMAD4* (3.8%), *BRAF* (2.9%), *NRAS* (2.9%), and *PIK3CA* (1.9%) were less common (< 10% of patients).

### Association between ctDNA and clinical factors

To estimate the clinical utility of ctDNA for patients with advanced pancreatic cancer, we sought to identify any associations between ctDNA and clinical factors. Because ctDNA was detected in 50% (52/104) of the cohort, we divided the patients into ctDNA-positive and -negative groups. With respect to age, gender, treatment at the time of sampling, primary site, with or without surgery, existence of lymph node metastasis, and CEA level, there were no significant differences between the two groups (Table 2). However, a higher number of liver metastases, the existence of lung and peritoneal metastases, a tumor diameter sum ≥ 100 mm, and a higher CA19-9 level were significantly associated with the detection of ctDNA (Table 2).

**Table 2:** Association of clinical factors with ctDNA in plasma.

Clinical characteristics	ctDNA negative	ctDNA positive	P value
Age at enrollment, y			0.59
Median	64	62	
Gender			0.41
Male	36	31	
Female	16	21	
Treatment line at sampling			0.61
None	0	1	
1^st^ line	20	18	
2^nd^ line	16	21	
3^rd^ line	11	11	
4^th^ line	5	1	
Pancreatic location of tumor			0.84
Head	27	29	
Body or tail	25	23	
Surgery at time of blood sample			0.34
Yes	14	9	
No	38	43	
Number of liver metastasis			<0.001
<10	37	19	
≥10	15	33	
Lymph node metastasis			0.12
Positive	30	45	
Negative	22	17	
Lung metastasis			<0.001
Positive	5	14	
Negative	47	38	
Peritoneal metastasis			<0.001
Positive	1	10	
Negative	48	45	
Sum of the tumor diameter (mm)			<0.001
<100	39	23	
≥100	13	29	
Tumor marker			
CEA median	5.8	8.8	0.081
CA19-9 median	368	4193	<0.001

ctDNA, circulating tumor DNA; CEA, carcinoembryonic antigen; CA19-9, carbohydrate antigen 19–9. RECIST ver 1.0 criteria.

### ctDNA as a prognostic marker in advanced pancreatic cancer with liver metastasis

**Fig 2:**
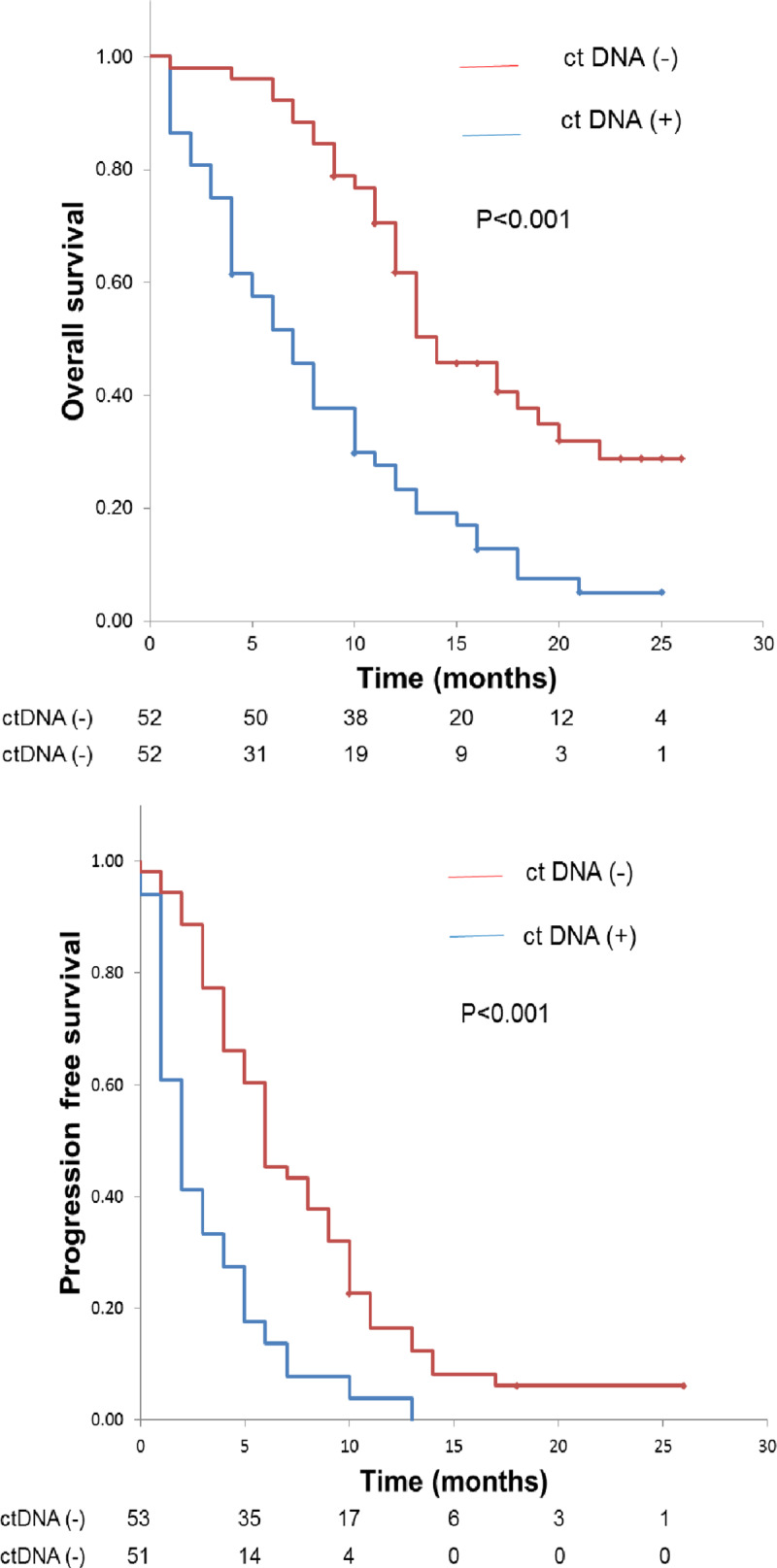
Kaplan–Meier estimates of overall survival and progression-free survival in pancreatic cancer patients with respect to the presence or absence of ctDNA. Comparison of overall survival (A) and progression-free survival (B) between ctDNA-positive and -negative groups. *P* values were calculated using the log-rank test.

We performed Kaplan–Meier analyses to assess the prognostic value of ctDNA in the plasma of patients with advanced pancreatic cancer. Using the log-rank test, we found a significant association between the presence (vs absence) of ctDNA and both OS and PFS (8.4 vs 16 months for OS and 3.2 vs 7.9 months for PFS for positive and negative, respectively, both *P* < 0.0001; Fig 2). Moreover, we performed univariate and multivariate Cox proportional hazard regression analyses, summarized in Table 3. In the univariate analysis, presence of ctDNA was significantly associated with OS (HR = 2.7, 95%CI = 1.7–4.2 months, *P* < 0.0001) and PFS (HR = 2.5, 95%CI = 1.7–3.8, *P* < 0.0001). Similarly, in the multivariate analysis, ctDNA was the only independent factor for OS (HR = 3.1, 95%CI = 1.9–5.0 months, *P* < 0.0001) and PFS (HR = 2.6, 95%CI = 1.7–4.0 months, *P* < 0.0001).

**Table 3:** Cox proportional hazard analysis for overall survival and progression-free survival in pancreatic cancer patients with liver metastasis.

Overall survival	Univariate analysis				Multivariate analysis			
	HR	Lower 95% CI	Upper 95% CI	P value	HR	Lower 95% CI	Upper 95% CI	P value
Variables								
Age (<65 or ≥65)	1.5	0.93	2.3	0.095	0.92	0.58	1.5	0.71
Gender	1.5	0.96	2.5	0.072	1.4	0.88	2.3	0.16
ctDNA (negative or positive)	2.7	1.7	4.2	<0.001	3.1	1.9	5.0	<0.001
CEA (<5 ng/ml or ≥5 ng/ml)	1.3	0.85	2.1	0.21	1.0	0.63	1.6	0.98
CA19-9 (<37 U/mL or ≥37 U/mL	1.3	0.75	2.2	0.37	1.2	0.70	2.0	0.61
Progression-free survival	Univariate analysis				Multivariate analysis			
	HR	Lower 95% CI	Upper 95% CI	P value	HR	Lower 95% CI	Upper 95% CI	P value
Variables								
Age (<65 or ≥65)	1.2	0.77	1.7	0.49	1.2	0.79	1.8	0.40
Gender	1.2	0.78	1.8	0.43	1.2	0.79	1.8	0.93
ctDNA (negative or positive)	2.5	1.7	3.8	<0.001	2.6	1.7	4.0	<0.001
CEA (<5 ng/ml or ≥5 ng/ml)	1.2	0.78	1.8	0.44	1.1	0.74	1.7	0.59
CA19-9 (<37 U/mL or ≥37 U/mL	1.2	0.72	1.9	0.53	0.90	0.55	1.5	0.69

ctDNA, circulating tumor DNA; CEA, carcinoembryonic antigen; Ca19-9, carbohydrate antigen 19–9.

### Association between ctDNA level and therapy response

Finally, we analyzed the association between therapy response after blood sampling and the presence or absence of ctDNA. The objective response rate (ORR) and disease control rate (DCR) were 9.6% (10/104) and 22.1% (23/104) (10 partial response [PR] and 23 stable disease [SD]), respectively. Patients with PR/SD after blood sampling showed better ORR than those with progressive disease ([PD], *P* = 0.001; Table 4).

**Table 4:** The association between ctDNA and objective response in patients with pancreatic cancer.

	PD	SD/PR	P value
ctDNA positive	41	7	0.001
ctDNA negative	30	26	

ctDNA: circulating tumor DNA. PD: progressive disease. SD: stable disease. PR: partial response.

## Discussion

Performing a comprehensive molecular analysis of tissue biopsy samples is generally one of the first steps in obtaining a diagnosis for cancer. However, the clinical utility of such an analysis is often diminished in patients with pancreatic cancer due to the difficulty in obtaining tissue samples of adequate quality [24]. Furthermore, taking biopsies can lead to clinical complications, which surgeons usually try to avoid if possible. As such, there has been considerable focus on the design and implementation of highly accurate and minimally invasive blood tests for cancer screening. Although several serological biomarkers, such as CEA and CA 19–9, are used in the clinic, they tend not to be sensitive or specific enough for prognostication in patients with pancreatic cancer [25]. More recent studies have shown that ctDNA—which can be obtained through liquid biopsies and is detectable by next-generation sequencing or ddPCR with high sensitivity—is released from the primary tumor and/or its metastases [26–29] and therefore could be used to detect cancer and/or its recurrence at very early stages. However, while several studies have described the clinical utility of ctDNA for patients with pancreatic cancer, few have used sufficiently large sample sizes for their analyses. In this study, we investigated the clinical utility of ctDNA as a biomarker for the prognostication of patients with advanced pancreatic cancer and liver metastasis.

Of the 104 patients enrolled in the present study, half had one or more detectable mutant allele(s) (MAF ≥ 0.15%) among 13 of 14 genes. This result is consistent with findings reported by Pietrasz and colleagues [30] and Kinugasa and colleagues [31]. However, Bettegowda and colleagues reported a much higher detection rate of ctDNA in patients with advanced pancreatic cancer [32]. These discordant results might be partially due to the differences in the number of genes included in the panel or the limit of detection, as follows. The molecular genetic landscape of pancreatic cancer has been studied by whole-genome and exome sequencing, and *KRAS*, *CDKN2A*, *TP53*, and *SMAD4* are frequently reported as mutated in pancreatic cancer [33–37]. In the current study, although *TP53* and *KRAS* mutations were detected in 90.4% and 84.6% of ctDNA-positive patients, respectively, few patients showed *SMAD4* mutations (3.8% of whole cohort, N = 4). These inconsistencies might be due to an insufficient coverage of mutations within the *SMAD4* gene in the panel; further technical improvement of the gene panel, which covers sufficient hot spot mutations of the *SMAD4* gene, would increase the detection accuracy in patients with pancreatic cancer. Another disadvantage of the cfDNA panel used in this study was that it did not contain *CDKN2A*, a common mutant gene in pancreatic cancer. Therefore, this study may have underestimated the number of ctDNA-positive samples. In the future, it would be better to measure ctDNA using a custom gene panel specific to pancreatic cancer. It was not possible to compare ctDNA with mutant DNA from tumor tissue in this study, as there were no sequence data on tumor tissue. Since ctDNA represents real-time tumor heterogeneity, it would be very useful in future to perform ctDNA analysis at the same time as mutant DNA analysis from biopsy tissues or surgical specimens.

We found a significant association of ctDNA with the number of liver, lung and peritoneal metastases, as well as with the sum of the tumor diameter, and the level of CA19-9, as previously reported [32, 38]. These factors may reflect the tumor burden better than other clinical factors. Since ctDNA has a short half-life, it can indicate the patient’s status including distant metastasis and tumor burden, in real time. Therefore, in the future, it may be possible to recommend a less invasive ctDNA test rather than an expensive CT test that increases risk of exposure, as has also been suggested by others [39, 40]. Lymph node metastasis was not significantly associated with the detection of ctDNA, suggesting that tumor burden in the lymph nodes might be relatively small compared with metastases in other organs. Clinical evidence suggests that metastasis to nearby lymph nodes occurs earlier in patients with pancreatic cancer than in those with other types of cancer, including liver, lung, and peritoneum. It is therefore not surprising that in the present study, almost all cases with liver metastasis also showed evidence of lymph node metastasis, and might explain why lymph node metastasis itself was not significantly associated with the presence of ctDNA.

We further showed that ctDNA was a prognostic marker of OS and PFS (OS: 8.4 vs 16 months with and without ctDNA, respectively; PFS: 3.2 vs 7.9 months with and without ctDNA, respectively; Fig 2; *P* < 0.0001). Our results agree with those of Pietrasz and colleagues, who previously reported the prognostic value of ctDNA as a biomarker in advanced pancreatic cancer [30]. In their study, the presence of ctDNA in the plasma correlated with poor PFS (9.9 months vs. median not reached; log-rank *P* = 0.02). Chen and colleagues also described an association between *KRAS* mutations detectable in the plasma and poor OS (3.9 vs. 10.2 months; *P* < 0.0001) [19]. Moreover, a meta-analysis of the association between cfDNA and the prognosis of pancreatic cancer patients concluded that certain mutations (KRAS, ERBB2-exon17, and KRASG12V), ctDNA presence, hypermethylation and higher concentration of cfDNA were associated with worse survival in patients with pancreatic cancer [10]. Reviews of the association between ctDNA and the prognosis of patients with pancreatic cancer suggest that high ctDNA levels have a poor prognosis, and that ctDNA is a more sensitive prognostic predictor than other tumor markers including CA19-9 [41, 42]. In our multivariate analysis, ctDNA was an independent predictor of OS (HR, 3.1; 95%CI, 1.9–5.0; *P* < 0.0001) and PFS (HR, 2.6, 95%CI, 1.7–4.0, *P* < 0.0001), further supporting the use of ctDNA as a reliable predictive biomarker in advanced pancreatic cancer. We also found a significant association between ctDNA and therapy response, which suggests that ctDNA could act as a biomarker to predict response to chemotherapy in patients with advanced pancreatic cancer. Periodic ctDNA analysis after the start of treatment may help determine whether to change treatment.

There were several limitations in our study. First, our study design was retrospective, and blood was collected at a single time-point which varied between patients. These variables might make it difficult to accurately analyze the association between ctDNA and various prognostic factors. In the future, a prospective follow-up and sequential blood sampling might be required to prove the utility of ctDNA as a biomarker. Second, of the genes which have a high mutation frequency in pancreatic cancer, KRAS, TP53, SMAD4, and CDKN2A, the frequency of mutations in SMAD4 were low and CDKN2A was not measured in this study. This may have led to underestimation of the number of ctDNA positives. In the future, a custom gene panel specific for pancreatic cancer should be used to accurately perform ctDNA measurement and further to compare mutated genes in paired tumor tissue and plasma samples.

In conclusion, we found that detection of plasma ctDNA can be used for prognostication in patients with advanced pancreatic cancer with liver metastasis. Future prospective studies could be undertaken to validate the clinical impact of ctDNA among this cohort and to confirm its clinical utility as a biomarker.

## Supporting information

S1 Table Somatic mutations detected by targeted sequencing of plasma cfDNA. Gene mutations in 14 genes from samples retrieved from 104 patients with advanced pancreatic cancer. White, no mutation detected; Gray, Mutant Allele Frequency < 0.15%; Yellow, 0.15% ≤ Mutant Allele Frequency < 1%; Blue, 1% ≤ Mutant Allele Frequency < 10%; Red, 10% ≤ Mutant Allele Frequency.
(XLSX)
